#  Preparation of Diaminedicarboxyplatinum (II) Functionalized Single-Wall Carbon Nanotube via Bingel Reaction as a Novel Cytotoxic Agent

**Published:** 2016

**Authors:** Saeed Irannejad, Mohsen Amini, Mona Modanlookordi, Mohammad Shokrzadeh, Hamid Irannejad

**Affiliations:** a *Department of Physics, University of Zanjan, Zanjan, Iran.*; b *Department of Medicinal Chemistry, Faculty of Pharmacy and Drug Design & Development Research Center, Tehran University of Medical Sciences, Tehran, Iran. *; c *Pharmaceutical Sciences Research Center, Faculty of Pharmacy, Mazandaran University of Medical Sciences, Sari, Iran. *; d *Department of Pharmacology & Toxocology, Pharmaceutical Sciences Research Center, Faculty of Pharmacy, Mazandaran University of Medical Sciences, Sari, Iran. *; e *Department of Medicinal Chemistry, Pharmaceutical Sciences Research Center, Faculty of Pharmacy, Mazandaran University of Medical Sciences, Sari, Iran.*

**Keywords:** ‎Singel-wall carbon nanotube (SWCNT)‎‎, Novel anti‎cancer agent, ‎Organoplatinum, Bingel reaction

## Abstract

Carbon nanotubes have unique properties like high stability, high surface to mass ratio and so on which make them suitable for medicinal purpose applications. Treatment of cancer by organoplatinum agents like Cisplatin has become unresponsive in most cases due to low distribution of drug in biological fluids, inability of drug to cross cellular membranes and low stability in biological environments. Recently, carbon nanotubes (CNT) have stimulated much interest to overcome these limitations. ‎‎ Herein, we report the preparation of single-wall carbon nanotube functionalized by diaminedicarboxyplatinum (II) as an analogy of SWCNT-based Carboplatin.

Functionalization was started by cyclopropanation through Bingel reaction and by use of diethylmalonate to yield cyclopropane-1,1-dicarboxy ethyl ester. Final product was obtained by hydrolysis of ester group and then chelation of platinum (IV) by dicarboxylate groups on the surface of SWCNT. Raman and Fourier transform -Infrared ‎spectroscopy (IR), ‎Thermogravimetric ‎analysis ‎(TGA) and energy dispers‎ive X-ray‎spectroscopy ‎(EDAX) truly showed and confirmed the presence of the platinum (II) complex on the side wall of SWCNT. Cytotoxicity evaluation of the functionalized-SWCNTs on HeLa cells showed its higher anticancer ability than Cisplatin as indicated by IC_50_ value of 13 µg/mL.

## Introduction

Single-wall carbon nanotubes (SWCNTs) are monolayer tubular nanostructures which are produced by some techniques including chemical vapor deposition (CVD), arc discharge and laser ablation. Since their discovery in 1993, there has been progressive interest among researchers for application of SWCNTs in numerous scientific fields.

To be applicable for medical purposes, CNTs have to be attached covalently or non-covalently to other molecules. To achieve this, chemical functionalization of SWCNTs would be inevitable. Functionalization of the CNTs is one of several ways utilized to improve the compatibility of CNTs. Carboxylate groups are one of the interesting and useful functional groups which are introduced on the sidewall of SWCNTs by strong oxidizing agents such as concentrated nitric or sulfuric acid. The produced carboxylate groups may be converted to the useful functional groups such as amides or esters to improve the CNT’s solubility (1).

Several studies have revealed the high penetration ability of CNTs through biological membranes and their application in drug delivery systems. Several medicinal or nutritional agents such as anticancer agents, metals and biomolecules have been loaded inside or attached on to the external surface of CNTs via chemical bonding or adsorbtion (2, 3).

CNTs have been extensively used for delivery of organoplatinum anticancer drugs into the tumor cells and tissues. Current platinum anticancer drugs have short half-lives in blood which tends to their rapid inactivation before their uptake in tumor cells (4).

**Figure 1:**
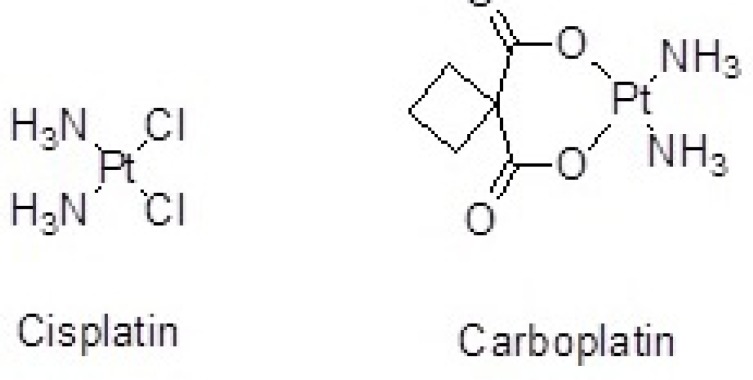
Organoplatinum anticancer agents: Cisplatin and Carboplatin

Cisplatin(*cis*-dichlorodiammineplatinum) (Figure 1.) is a platinum-containing antineoplastic agent applied to treat a wide variety of malignant tumor cells. Drug resistance, instability in aqueous solution, short half-life, inability to cross cellular membranes and reduced cellular uptake limit the effectiveness of Cisplatin.

In this situation, Carbon nanotubes have been a promising scaffold for improving biodistribution and prolonging blood circulation of unstable agents. They can carry various loaded or attached chemicals into the cells via Clathrin mediated endocytosis that would not otherwise be taken up by cells. Functionalized carbon nanotubes by suitable chemical modifications can endure more time in blood circulation with longer half-lives. As a consequence, various molecules linked on to the surface or loaded into the inner space of SWCNTs can be internalized easier (5, 6).

Encapsulation of small drug molecules within SWCNTs as a drug delivery system have been performed by many investigators (7, 8).

Several studies have been used SWCNTs as a drug delivery vehicle for Cisplatin in cancer cell lines. Guven *et al*. encapsulated Cisplatin inside ultra-short carbon nanotubes (US-CNT) and evaluated the cytotoxicity of the prepared nanocapsules. Results indicated in high accumulation of Cisplatin nanocapsules inside tumor cells and increase in cytotoxicity of Cisplatin (9).

In another study, organoplatinum drug attached to the surface of SWCNTs by covalent bond (peptidic bond) increased stability of drug and penetration to the tumor cells (10). Cisplatin has also been loaded inside carbon nanohorns as a drug delivery nanocontainer to prevent rapid deactivation and decomposition and to prolong its circulation time in blood (11). Similar studies have been performed by the use of CNTs as nanocarriers of Carboplatin. Alkylating antineoplastic agent Carboplatin (diammineplatinum(II)cyclobutane-1,1-dicarboxylate) is a newer platinum agent with higher water solubility and fewer side-effects than the older one, Cisplatin (Figure 1.) (12, 13).

**Figure 2.**
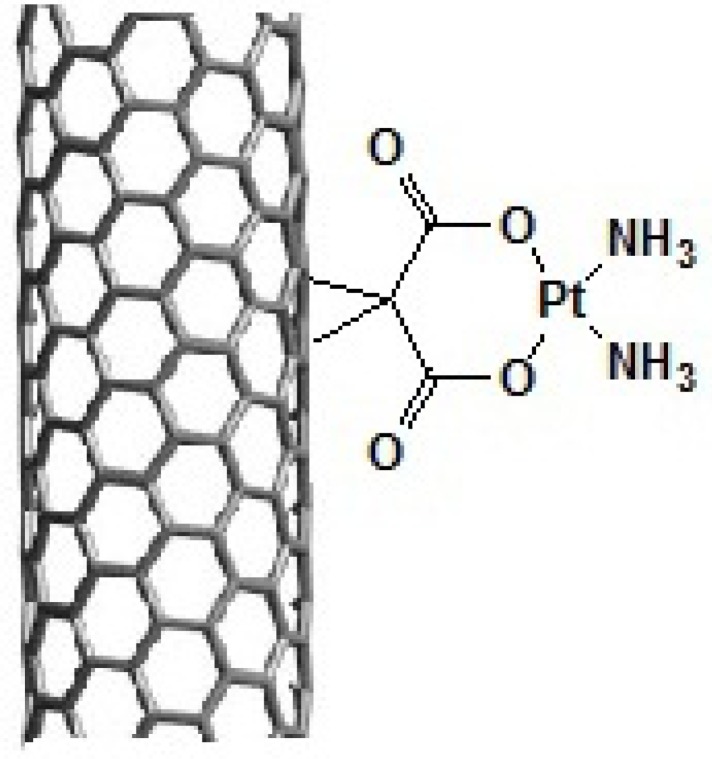
Schematic representation of the prepared Diammineplatinum (II) cyclopropan-1,1-dicarboxylate-SWCNT (*dcd-SWCNT*) as a novel anticancer agent

In this study, we have covalently attached organoplatinum (II) on the sidewall of SWCNT. The structure of organoplatinum formed is diammineplatinum (II) cyclopropan-1,1-dicarboxylate-SWCNT (*dcd-SWCNT*) and structurally similar to Carboplatin (Figure 2.). SWCNTs were first functionalized by Bingel reaction, a [2+1] cycloaddition reaction, to produce cyclopropan-1,1-dicarboxylate and then diamineplatinum (II) was chelated by dicarboxy groups to form the final nanoorganoplatinum (II). Formation of the final structure was assessed and confirmed by Raman and Fourier transform -Infrared ‎spectroscopy (IR), ‎Thermogravimetric‎ analysis ‎(TGA) and energy dispers‎ive X-ray‎spectroscopy ‎(EDAX). Finally, cytotoxic evaluation of the nano-product was assessed on HeLa cells (cervical carcinoma cells) to examine its anticancer potential.

*Methods*

Solvents and chemical reagents including Cisplatin were purchased from Sigma-Aldrich. Single-wall carbon nanotubes were produced by CVD method. The purity of samples was evaluated via thermogravimetric analysis (TGA) (air ﬂow, heating ramp 10 °C/min to 1000 °C, DTA) using a STA-503 analyzer (Germany). Raman spectroscopy (SENTERRA, BRUKER Raman Microscope Spectrometer) was employed to ensure the functionalization of SWCNT sidewall (laser = 785 nm). Infrared spectra were recorded on a Perkin-Elmer instrument to characterize the carbonyl functional groups. SEM electron microscopy and energy-dispersive X-Ray analyzer (VEGA TESCAN, Czech Republic) were used to investigate the appearance and chemical composition of functionalized SWCNTs.

*Synthetic procedure*

To remove the metallic impurities and non-nanotube carbon materials, sample was refluxed for 24 h in a concentrated nitric acid solution, and then ultrasonicated for 5 h in a mixture of HNO_3_:H_2_SO_4_ solution (1:2). The acid treatment also cuts SWCNTs to yield shortened SWCNTs, which have carboxylic groups at the open ends and defect sites (14).

*Bingel reaction was performed as following procedure*

*Preparation of SWCNT-cyclopropan-1,1-dicarboxylate ethyl ester*

Acid-treated SWCNTs (50 mg) were suspended in 5.0 mL of dry tetrahydrofuran by ultrasonication in a 100 W sonic water bath for 30 min. To this suspension were added Diethylmalonate (100 mg, 0.33 mmol) and iodine (85 mg, 0.33 mmol), then 1,8-diazabicyclo[5.4.0]undec-7-ene (DBU) (0.10 mL, 0.67 mmol) was added dropwise and the mixture stirred vigorously for 24 h under nitrogen atmosphere. After completion of the reaction time, the reaction mixture was filtered with a 0.22 μM pore-size membrane filter and washed with chloroform and DI water to remove the unreacted materials and impurities (15, 16).

*Hydrolysis of SWCNT-cyclopropan-1,1-dicarboxylate ethyl ester*

Hydrolysis was performed by adding 5 mL of 1M NaOH to the previous step product in methanol and stirring continued for 24 h. Then, the mixture was filtered with a 0.22 μM pore-size membrane filter, washed by deionized water and kept under nitrogen atmosphere (17).

*Preparation of Diammineplatinum (II) cyclopropan-1,1-dicarboxylate-SWCNT (dcd-SWCNT):*

Cisplatin (60 mg) was added to Silver nitrate (70 mg) solution in deionized water (50 mL) and warmed to 60 °C on a hot plate with stirring until the silver chloride precipitation was complete. Precipitated silver chloride was filtered off using a fine pore filter and the precipitate was washed several times with hot deionized water. The filtered mother liquor was almost colorless (18). This transparent solution was added to SWCNT-cyclopropan-1,1-dicarboxylatein deionized water (pH = 5-6) and the mixture stirred at 60 °C for 2 h. Finally, the reaction mixture was filtered on a 0.22 μM pore-sized filter and washed several times with hot deionized water to give the final product (15 mg). 

*Cell line and viability assay (MTT assay)*

Cytotoxicity was determined with human cervical carcinoma epithelial cells HeLa. Cells were cultured in 75 mL flasks in RPMI, supplemented with the antibiotic solution (1% of penicillin/streptomycin stock), glutamine, NaHCO3 and 10% fetal bovine serum (FBS) at 37 °C in a humidified atmosphere of 5% CO_2_. The medium was changed every two days and the cells were subcultured after reaching confluence.

In MTT assay, 50 μL of RPMI including 1×10^5^ cells were added to 3 wells for each concentration of *dcd-SWCNT* and Cisplatin as reference drug. Then, they incubated for 72 h. After incubation, the cell lines were exposured to 50 μL of each 0, 1, 50, 100, 500 and 1000 μg/mL of *dcd-SWCNT* and 10 μM of Cisplatin for 72 h, and then washed using sterile normal saline 0.09%. After this period, the contents of wells were excluded; the cells were dyed by 30 μL of MTT (Methyl thiotetrazolium) and incubated for 4 h. Then, MTT solution was excluded from wells, and 30 μL of DMSO was added to each well and the 96 well/plates were shaked for 15 min. At the end, the absorbance was determined by ELISA reader (λ max = 490 and 630 nm). 

*Statistical analysis*

GraphPad Prism 5 Software was used to perform statistical tests. Analysis of variance (one-way ANOVA) followed by Dunnett test was used to compare the significance of groups vs. Cisplatin 10 µM as control group (p<0.05).

## Result and discussion

In this study, Bingel reaction, a [2+1] cycloaddition reaction, was used for the functionalization of ‎singel-‎wall carbon nanotubes (SWCNT). Bingel reaction is one of the most frequently employed methodologies for the functionalization of fullerenes due to the high reactivity under mild reaction conditions (19).

**Figure 3:**
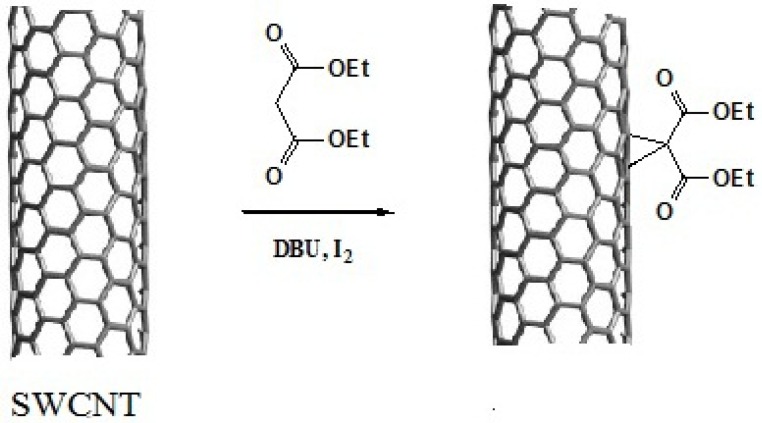
Schematic presentation of Bingel reaction on the sidewall of SWCNT.

**Figure 4:**
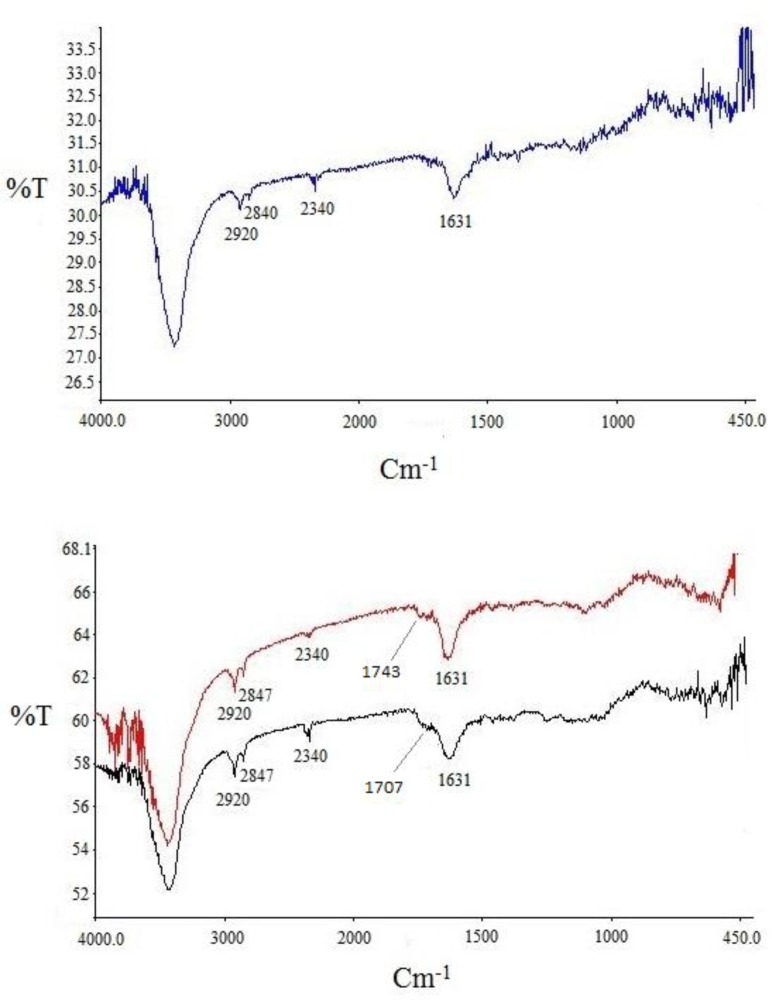
FT-IR spectra for purchased SWCNT (a, blue curve), acid-treated SWCNT (b, black curve) and SWCNT-cyclopropan-1,1- dicarboxylate ethyl ester (b, red curve

**Figure 5:**
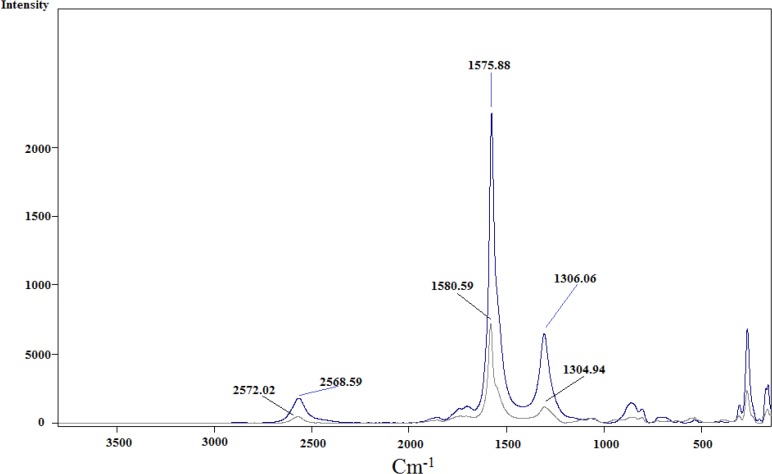
Raman spectra for acid-treated SWCNT (blue) and SWCNT-cyclopropan-1,1-dicarboxylate ethyl ester (gray

**Figure 6:**
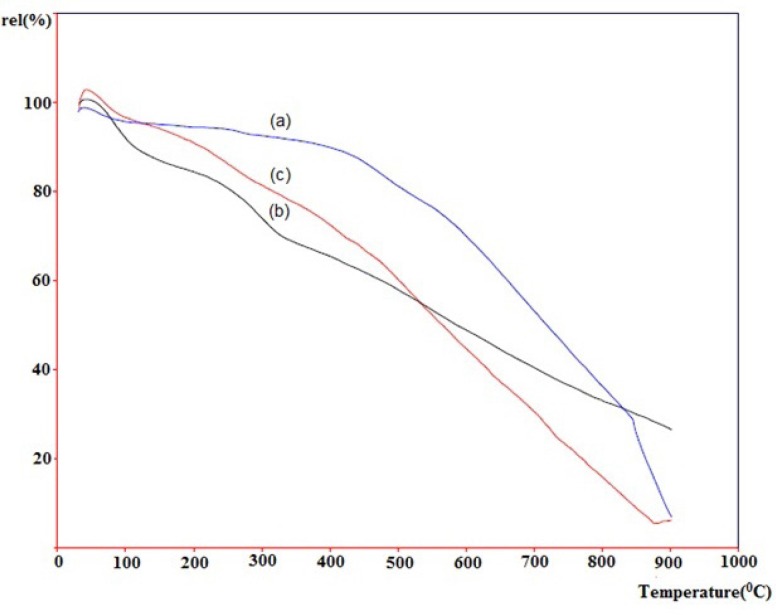
TGA thermogram of pristine SWCNT (a, blue), acid-treated SWCNT (b, black) and SWCNT-cyclopropan-1,1-dicarboxylate ethyl ester (c, red

**Figure 7.**
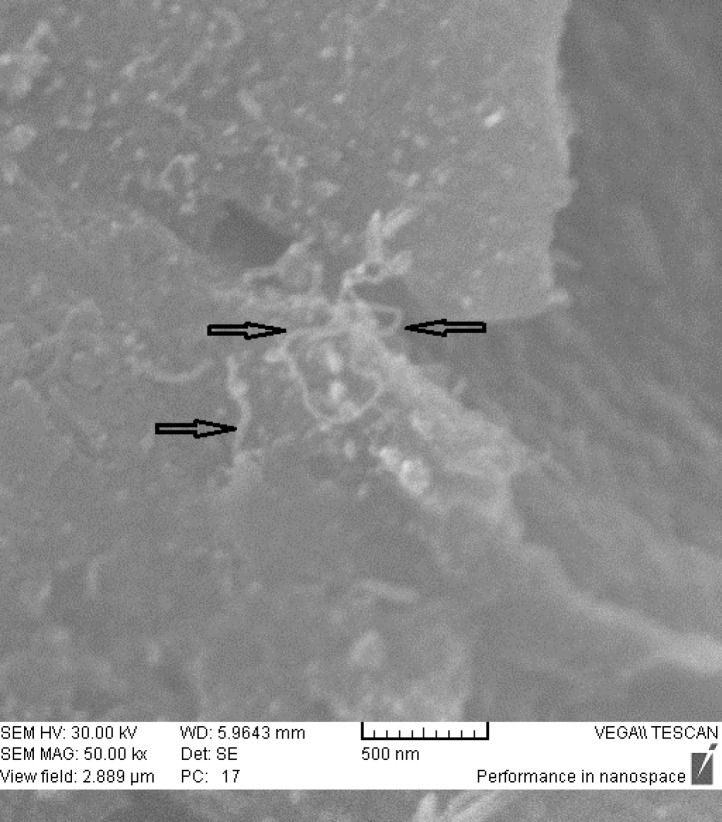
SEM images of *dcd-SWCNT*.

**Figure 8:**
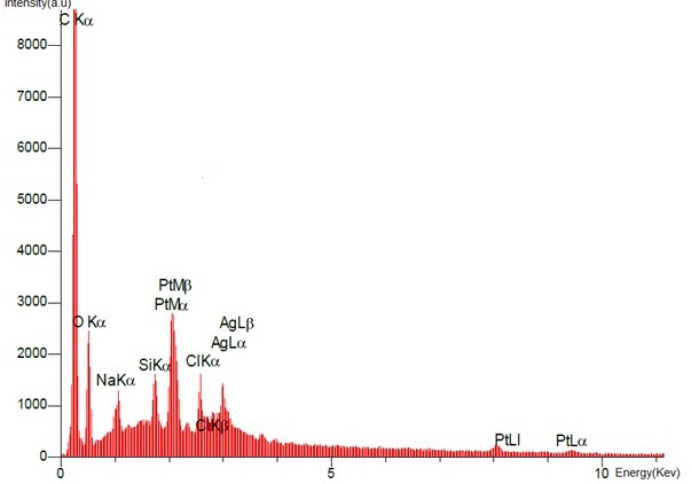
EDX spectrum of the final product, Diammineplatinum (II) cyclopropan-1,1-dicarboxylate-SWCNT (*dcd-SWCNT*) is presented. Signals from Carbon (from nanotubes) and platinum (from the chlated platinum atom) have been detected.

**Figure 9:**
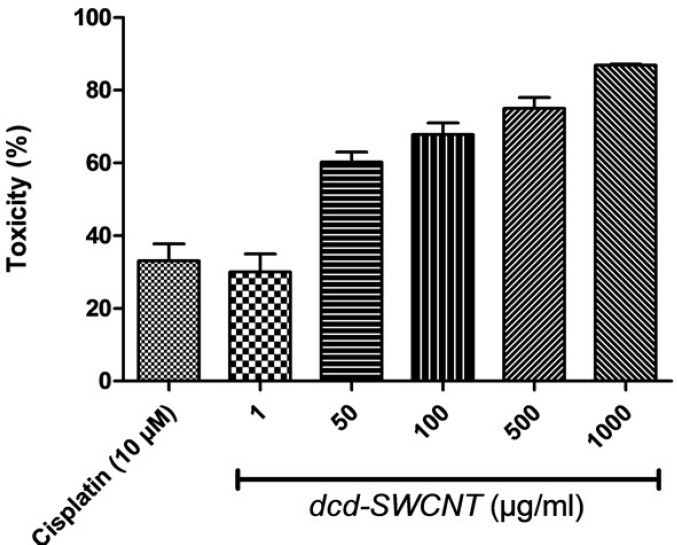
Cytotoxicity evaluation of *dcd-SWCNT* at different concentrations (µg/mL) and the reference drug cisplatin(3 µg/mL, 10 µM).

**Scheme 1:**
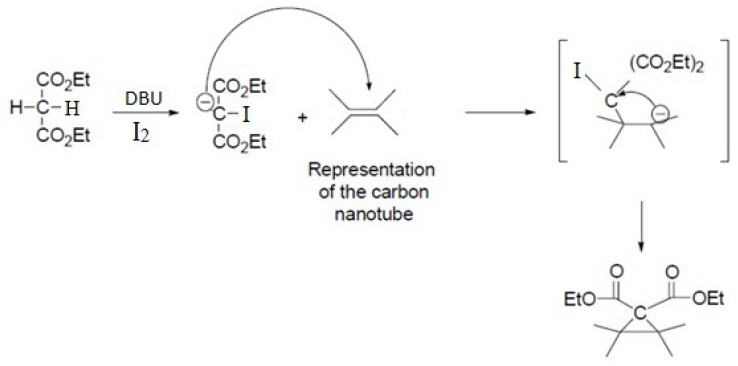
Schematic representation of Bingel reaction on an isolated double bond of carbon nanotube

**Table 1:** Cytotoxic activity (%) of the synthesized *dcd-SWCNT* in five different concentrations on HeLa cells

**Compound (Conc.)**	**%Toxicity ± SEM**
isplatin (10 µM)	33.05±4.6
dcd-SWCNT (1 µg/ML)	30.0±4.9
dcd-SWCNT (50 µg/ML)	60.2±2.7***
dcd-SWCNT (100 µg/ML)	67.83±3.1***
dcd-SWCNT (500 µg/ML)	75.0±2.9***
dcd-SWCNT (1000 µg/ML)	86.83±0.29***

The results are the mean of three determinations.

*** P< 0.0001

This reaction proceeds with the breakage of π -bonds in the benzene ring of carbon nanotube and concomitant formation of new σ-bonds with diethylmalonate in the presence of iodine and diazabicycloundecene (DBU) to induce the formation of a cyclopropane-1,1-dicarboxylate ethyl ester group on the surface of SWCNT (Scheme 1. and Figure 3.) (20). In the next step, dicarboxylate ethyl ester group was hydrolyzed with sodium hydroxide to give the free acid. In parallel, silver nitrate was added to Cisplatin (cis-‎Diamminodichloroplatinum) in order to precipitate silver chloride and activate the organoplatinum (II) as shown in the following formula.

[Pt(NH_3_)_2_Cl_2_]+2AgNO_3_+2H_2_O→[Pt(NH_3_)_2_

(H_2_O)_2_](NO_3_)+2AgCl

Finally, activated organoplatinum was added to the SWCNT-cyclopropan-1,1-dicarboxylate. At this step, a complex forms with the chelation of platinum (II) by dicarboxylate anion on the sidewall of SWCNT.

The functionalized SWCNTs via Bingel reaction detailed above were analyzed using Fourier Transform Infrared Spectroscopy (FT-IR), Thermogravimetric analysis (TGA), Raman analysis and energy-dispersive X-Ray analyzer (EDX).

*Characterization of FT-IR spectra*

Infrared analysis was applied to detect the functional groups formed during functionalization over successive steps. Comparison of the IR spectra of carbon nanotubes before and after modification could reveal whether new bonds were formed. Three spectra were recorded and are shown in Figure 4. for pristine SWCNT in blue, acid-treated SWCNT in black and cyclopropanated SWCNT in red. Changes in the spectra after acid-treating and formation of carboxyl groups and after Bingel reaction to form ester functions are well characterized.

In acid-treated SWCNT (spectrum in black), presence of new band at 1707 cm^-1^ indicates C=O stretching of carboxylate. The IR spectrum of the Bingel reaction product exhibits C=O stretching of the ester group (-C(=O)-OEt) at 1743 cm^-1 ^which supports the sidewall functionalization and formation of cyclopropan-1,1-dicarboxylate ethyl ester.

*Thermogravimetric Analysis (TGA)*

Thermogravimetric analysis (TGA) has been widely used to investigate the covalent functionalization of SWCNTs. Figure 6. depicts thermograms of pristine-SWCNT (a, blue line), acid-treated SWCNT (b, black line) and the cyclopropanated SWCNT after Bingel reaction (c, red line) measured in air. Horizontal axis shows increase in temperature until 1000 °C and vertical axis shows the relative weight loss. Normally, materials lose weight as temperature increases due to separation of chemical entities or decomposition in higher temperatures.

The weight loses in the temperature ranges of 200-400 and 400-750 °C are assigned to the detachment of the covalently linked substituent and the combustion of SWCNT, respectively. As shown, pristine-SWCNT has the most thermal stability which does not have any functional group. Rate of weight loss in acid-treated SWCNT is highest until 400 °C due to the easy detachment of carboxyl and hydroxyl groups on the sidewall of CNT. Rate of the weight loss in cyclopropanated-SWCNT is slower than acid-treated SWCNT until 500 °C but becomes more rapid above this temperature. 

This type of reduction in weight is consistent with the previously performed studies in functionalized CNTs (16, 21).

*Energy Dispersive X-ray Analysis*

SEM images of the final product *dcd-SWCNT* are shown in Figure 7. and show two isolated SWCNTs. In Figure 8. the EDX spectrum presents the elemental analysis and chemical characterization of the final product, Diammineplatinum (II) cyclopropan-1,1-dicarboxylate-SWCNT (*dcd-SWCNT*). Peaks corresponding to the characteristic carbon (fromnanotubes), platinum (from chelated platinum) are detected. Some unwanted peaks are also present for sodium, silver, silisium and chlorine atoms. The one related to chlorine atom is probably due to the unreacted Cisplatin that is adsorbed on the surface of SWCNTs or is related to the silver chloride impurity whereas silver atom peak is also present.

*In-vitro cytotoxicity assay*

Cytotoxic activity of the synthesized *dcd-SWCNT* was evaluated on HeLa cells which are cervical carcinoma epithelial cells and compared with the reference drug Cisplatin and the results with SEM and P values are summarized in Table 1. The reference drug, Cisplatin showed 33% toxicity at 10 µM or 3 µg/mL. Surprisingly, *dcd-SWCNT* showed higher cytotoxicity over Cisplatin in which 30% toxicity was obtained at 1 µg/ml of *dcd-SWCNT*. 

Toxicity evaluation at several concentrations of *dcd-SWCNT* revealed its IC_50_ value of 13 µg/mL (Figure 9.). Since the extent of side wall functionalization of SWCNT is relatively low according to literature (one diester unit per ~100 carbon atom on the sidewall) (16), therefore, toxicity observed in higher order of magnitude than cisplatin could be attributable to its high permeation capability of *dcd-SWCNT *into the cell membranes and its higher stability in aqueous biological environments (22).

## Conclusions

A detailed methodology for the modification and functionalization of Single-wall carbon nanotube via Bingel reaction, a [2+1] cycloaddition, has been presented. Platinum (II) was chelated by dicarboxylate group of SWCNT-cyclopropan-1,1-dicarboxylate to give the final product. Diammineplatinum (II) cyclopropan-1,1-dicarboxylate-SWCNT could be a novel and potential anticancer agent. This SWCNT-based organoplatinum might have high blood circulation time, high cell permeation and more stability over Cisplatin. FT-Infrared (FTIR) spectroscopy, Raman spectroscopy, Thermal analysis (TGA–DTA) and EDX analysis data have shown the presence of the cyclopropan-1,1-dicarboxylate group and platinum atom attached to the surface of SWCNT. Further study on the anticancer evaluation of this nano-product revealed its higher cytotoxic potential than the parent drug cisplatin.

*Competing interests*

The author(s) declare that they have no competing interests.

*Authors´ contributions*

SI has contributed in the preparation and characterization of functionalized-SWCNTs, MA contributed in the technical assessment and interpretation of results. MM and MS performed in vitro cytotoxicity evaluation and HI contributed as the leadership and designing of experiments and synthetic procedures.
